# A Structural Competency Curriculum for Primary Care Providers to Address the Opioid Use Disorder, HIV, and Hepatitis C Syndemic

**DOI:** 10.3389/fpubh.2020.00210

**Published:** 2020-06-05

**Authors:** Ann D. Bagchi

**Affiliations:** Rutgers School of Nursing, The State University of New Jersey, Newark, NJ, United States

**Keywords:** structural competency, syndemic, curriculum, primary care, opioid use disorder, HIV, hepatitis C

## Abstract

The interrelated epidemics of opioid use disorder (OUD) and HIV and hepatitis C virus (HCV) infection have been identified as one of the most pressing syndemics facing the United States today. Research studies and interventions have begun to address the structural factors that promote the inter-relations between these conditions and a number of training programs to improve structural awareness have targeted physician trainees (e.g., residents and medical students). However, a significant limitation in these programs is the failure to include practicing primary care providers (PCPs). Over the past 5 years, there have been increasing calls for PCPs to develop structural competency as a way to provide a more integrated and patient-centered approach to prevention and care in the syndemic. This paper applies Metzel and Hansen's (1) framework for improved structural competency to describe an educational curriculum that can be delivered to practicing PCPs. Skill 1 involves reviewing the historical precedents (particularly stigma) that created the siloed systems of care for OUD, HIV, and HCV and examines how recent biomedical advances allow for greater care integration. To help clinicians develop a more multidisciplinary understanding of structure (Skill 2), trainees will discuss ways to assess structural vulnerability. Next, providers will review case studies to better understand how structural foundations are usually seen as cultural representations (Skill 3). Developing structural interventions (Skill 4) involves identifying ways to create a more integrated system of care that can overcome clinical inertia. Finally, the training will emphasize cultural humility (Skill 5) through empathetic and non-judgmental patient interactions. Demonstrating understanding of the structural barriers that patients face is expected to enhance patient trust and increase retention in care. The immediate objective is to pilot test the feasibility of the curriculum in a small sample of primary care sites and develop metrics for future evaluation. While the short-term goal is to test the model among practicing PCPs, the long-term goal is to implement the training practice-wide to ensure structural competence throughout the clinical setting.

## Introduction

In 2017, an estimated 47,600 Americans died from opioid overdoses, representing 67.8% of all drug-related overdose deaths that year (2). Vital statistics demonstrate significant overlap in the opioid misuse epidemic with infectious disease outbreaks, with the most recent estimates suggesting that ~67% people who inject opioids are infected with the hepatitis C virus (HCV) and 33% with HIV (3, 4). Furthermore, in the 2014–2015 HIV epidemic in Scott County, Indiana, among the 181 people newly diagnosed with HIV, 88% had injected oxymorphone and 92% were co-infected with HCV (5).

These interrelated and synergistic relationships between epidemics has been defined as a “syndemic,” (6) reflecting temporal, geographical, and biological interactions between the individual disorders. The syndemic of opioid use disorder (OUD) and overdose, HIV infection, and HCV infection (hereafter, “the syndemic”) has been appropriately identified as one of the most pressing public health issues facing the United States today (7). As Milstein has described, addressing this, or any syndemic, requires prevention, and treatment of each individual problem, as well as “the forces that tie those diseases together” [(8), p. 2]. Chief among these is the need to combat structural stigmas that have led to siloed and inefficient systems of care (e.g., specialty addiction treatment facilities to manage OUD, infectious disease [ID] specialists/the Ryan White HIV/AIDS Program for HIV, and gastroenterology/ID to manage HCV and associated chronic liver disease).

While Goffman (9) is generally cited as providing the earliest treatise on stigma, more recent theorists have expanded on his original ideas to advance a more nuanced understanding of how stigma operates in the United States (9). The model of Link and Phelan (10) is particularly suitable for understanding the syndemic because it identifies labeling, stereotyping, prejudice, and discrimination as the major components of stigma, but also highlights the role that power plays in perpetuating stigma (10). In this conception, societal structures (e.g., economic, social, political, and historical systems) create inequitable systems of power that enable expressions of stigma, which then create and sustain health inequities. As such, eliminating stigma requires moving beyond individual-level interventions (e.g., behavioral treatment for substance use disorder and reducing rates of opioid prescribing) to higher-level disruptions in systemic and structural factors that perpetuate health inequities.

### The Role for Primary Care Providers (PCPs)

A commonly-cited barrier to addressing the syndemic has been the lack of access to prevention and treatment services (11–14). Estimates suggest that among the 22 million people in need of addiction services, only 11% have access to specialty care and, among those with OUD, almost 80% lack access to treatment (11, 12). In the HIV epidemic, despite long-standing recommendations from the CDC that all individuals ages 13–64 receive an HIV test (15) only 40–46% of adults have ever received one (16, 17) and, in 2018, only 8.1% of individuals at high-risk for infection received pre-exposure prophylaxis (PrEP) (18). Finally, the CDC estimates that nearly 2.4 million people are living with HCV in the United States and notes that the cost of treatment has led to underutilization of curative therapy (19). The United States Preventive Services Task Force (USPSTF) recently posted updated recommendations for HCV testing (20). The previous recommendation was that all adults born between 1945 and 1965 be screened for HCV, but the new guidelines suggest screening for all adults ages 18–79. Although there have been successful models of care integration for management of HIV and OUD, these have largely been in specialty care settings and there has been inadequate uptake in primary care practices (21). Similarly, while there have been successful models of HCV integration into primary care (22–24), including increasing consideration for OUD (25), such comprehensive approaches remain underutilized.

As a way to address the opioid overdose epidemic, several state- and federally-funded initiatives have focused on more responsible opioid prescribing among primary care providers (PCPs), including daily limits on milligrams of morphine equivalents (MME) and mandatory consultation of prescription drug monitoring program (PDMP) databases. However, as Dasgupta et al. (26) note, these approaches ignore institutionalized racial biases (e.g., laws that have criminalized drug use by members of ethnic minority groups as reflecting individual decision-making and “moral failures”) and structural factors (e.g., poverty and limited social capital) that underlie components of the syndemic (26). In addition, these opioid-specific approaches perpetuate the siloed nature of health services and fail to take the type of integrated approach that is needed to combat the syndemic. For example, the focus on opioid-related overdoses frequently overlooks other co-occurring substance use disorders, such as injection of methamphetamines, which also contributes to increase risk for HIV/HCV and overdose risks associated with polypharmacy (e.g., opioids and sedatives or stimulants) (27–30).

Providers in primary care settings (e.g., private practices, federally qualified health centers, and retail clinics) are uniquely positioned to offer comprehensive, patient-centered care that can accommodate individual needs. Greater incorporation of guideline-based screening into existing care and services will allow practicing PCPs to address service gaps without necessitating significant changes in clinic workflow or operations (14). Most importantly, by normalizing prevention and treatment services in primary care settings, the healthcare system can begin to address the stigma that underlies the critical intersection of the disorders within the syndemic. In general, PCPs can take a more active role in addressing structural stigmas and there have been increasing calls over the last 5 years for PCPs to develop structural competency as a way to provide a more integrated and patient-centered approach to prevention and care in the syndemic (1, 7, 31, 32). In particular, structural competency is seen as a way to address the institutionalized factors that shape social responses and clinical interactions (1). Recent reviews highlight successful office-based models for treatment of substance use disorders, which can be modified to address structural factors fueling the syndemic (13, 21, 33). For example, harm reduction programs and medication for addiction treatment (MAT) promote a patient-centered approach to treatment that shows promise for overcoming barriers associated with socioeconomic status, institutionalized racism in the criminal justice system, and stigma.

### The Model for Structural Competency Training

In their seminal paper, Metzel and Hansen (1) defined *structural competency* as “the trained ability to discern how a host of issues defined clinically as symptoms, attitudes, or diseases … also represent the downstream implications of a number of upstream decisions” related to public policies, supply chains operating within the healthcare system, and even “the very definitions of illness and health” (32, p., 5). The authors propose the need to expand traditional models of “cultural competency” into an educational approach that recognizes, and seeks to interrupt, these long-standing interactions, which perpetuate stigma and social inequality. As with the construct of “cultural humility,” (34) structural competency is understood not as an endpoint denoting mastery, but as a process of genuine self-reflection and recognition.

#### “Recognizing the Structures that Shape Clinical Interactions” (32, p. 6)

Metzl and Hansen (1) described five skill-sets to form the basis for a structural competency curriculum for health care professionals (1). The first calls on clinicians to recognize the ways in which patient-provider interactions operate as functions of structural vulnerability. When a patient presents with poorly controlled diabetes, providers may assume that the patient (a) is reluctant to exclude “culturally preferred” foods from their diet (b) needs additional education on insulin administration, or (c) simply lacks the motivation to participate in recommended physical activity. Indeed, guidelines for management of such chronic conditions as diabetes and hypertension highlight “lifestyle changes” as the preliminary approach to management. However, such assumptions overlook factors associated with inadequate access to fresh foods and unsafe neighborhoods that restrict opportunities for daily exercise. While there is increasing awareness of these social determinants of health, structural factors, such as the pressure to conform to a 15-min encounter or the policies related to drug reimbursement, while recognized, go relatively unchallenged. It is important for clinicians to recognize that the social determinants of health may be the factors that contribute to disparities, but it is the structural conditions within society that explain why and how these factors lead to health inequities (35).

A common barrier to guideline-based screening in primary care settings is a belief that incidence of a given condition (e.g., HIV) is low in the provider's service area (36). Such attitudes perpetuate the ineffective, siloed approach to primary care practice. Van Handel et al. (37) found that six factors of structural vulnerability are predictive of the risk for syndemic outbreaks within a given geographic area (37). Specifically, these indicators include (a) the overdose death rate; (b) the number of prescription opioid sales; (c) the capacity for buprenorphine administration, as evidenced by the number of providers with a Drug Addition Treatment Act of 2000 (DATA 2000) waiver; (d) the percentage of non-Latino white residents; (e) per capita income; and (f) the unemployment rate. PCPs should consider these broader conceptions of risk in the provision of preventive services, including screening for HIV and HCV among patients receiving opioid prescriptions.

#### “Developing an Extra-clinical Language of Structure” (32, p. 7)

The second skill of Metzel and Hansen's (1) framework challenges PCPs to consider structural barriers from the perspective of other disciplines (e.g., psychiatry, public health, sociology, and anthropology) (1). Abundant evidence documents ethnic health disparities in infant mortality rates, obesity, and cancer screening (38–40). However, a structurally competent approach explores the nature of these disparities in their historical, economic, and sociological context. For example, Ransome et al. (41) explored the structural factors leading to late presentation for HIV testing (i.e., when infection has already progressed to AIDS) in communities with high concentrations of African American residents (41). These authors found that high socioeconomic deprivation and access to testing services did not mediate the association. They suggested the need to consider patterns of marriage/sexual partnerships and disproportionate incarceration rates as factors underlying diagnostic disparities.

#### “Rearticulating ‘Cultural' Presentations in Structural Terms” (32, p. 9)

The third skill in Metzel and Hansen's framework requires providers to reframe “cultural differences” in terms of structural explanations (1). In their various publications, Metzl and Hansen draw a clear distinction between cultural and structural competency. They describe cultural competency as a process that operates at the individual level to identify clinicians' biases and to enhance patient-provider communication (42). Structural competency, on the other hand, is a method of integrating explanatory frameworks from multiple disciplines to identify higher level sources of health inequities (42, 43).

In a clinical scenario they present, Metzl and Hansen (1) describe Mrs. Jones as “an African American woman in her mid-60s who comes late to her office visit and refuses to take her blood pressure medications as prescribed” (32, p. 2). Under a cultural competency framework, the clinician may see the patient's ethnicity as a source of greater susceptibility to hypertension. The fact that she “comes late” to her appointment could be dismissed as a cultural proclivity against the value of timeliness. Finally, her “[refusal] to take” her prescribed medications may indicate to the clinician a need to provide more patient education regarding the effects of hypertension on critical organ systems and the importance of medication adherence for maintaining a healthy blood pressure. While it is important not to discount such factors in a clinical encounter, a structurally competent approach would consider, for example, how systemic structural racism can lead to a sense of hypervigilance among members of ethnic minority groups and that such a constant state of awareness leads to stress, which can in turn lead to increased blood pressure levels. The structurally competent approach moves beyond “genetic” and individual factors and considers how society operates to reinforce racial injustice.

#### “Observing and Imagining Structural Intervention” (32, p. 10)

In the traditional approach to care, clinicians would consider the case of Mrs. Jones and might provide her with a pill organizer or set up text or telephone reminders for her to take her medications. Most primary care practices abound with patient-facing educational brochures to explain common, chronic health conditions and how to better manage them. However, a structurally competent approach to care requires creativity and a willingness to disrupt long-standing assumptions about what is possible in clinical care (Skill 4). As Metzl (44) eloquently put it, structural forces should not be seen as “immutable or beyond the reach of intervention or repair” but as “stories” that are “subject to revision through imagination, reparation, and transformation” (44, p. 217). As Metzl and Hansen (1) point out, Dr. Jack Geiger started prescribing food as a health intervention in the 1960s (45). At the time, such an approach was seen as unusual and impractical. Today, food prescriptions have become a common practice for managing obesity, hypertension, and diabetes (46).

#### “Developing Structural Humility” (32, p. 12)

The final component to a structurally competent approach is developing an openness to a patient's evolving narrative (1). The American Academy of Pediatrics is credited with coining the concept of the patient-centered medical home in 1967 and “patient-centeredness” has been an idealized notion in the healthcare system for the past 50 years (47). However, it is rare to find a health care delivery system that treats the patient as a true co-equal collaborator in their own care. Many people who have served as the health caretaker of someone unable to speak for themselves can relate to the experience of being dismissed by a member of the medical establishment (i.e., “We can't find anything wrong with your son/mother/brother/etc.”). However, the caretaker often knows when their loved one is “not acting right.” A clinician taking a structurally competent approach solicits the patient/caregiver's insights as a co-equal “expert” on the patient's condition as part of everyday practice.

According to Montoya (48), there are four keys to structural humility (48). The first reflects this view of patients as authorities and calls on clinicians to ask “real questions,” ones “for which you do not already have an answer” (48, p. 153). For example, Kleinman's Explanatory Models Approach (49) solicits the patient's narrative (e.g., “What do you call this problem?,” “What do you believe is the cause of this problem?”), rather than simply accepting the biomedical model, which assumes the provider already knows the answers relating to the problem's description and etiology. The second is to embrace discomfort. In describing Yale's Department of Psychiatry Structural Competency Community Initiative (YSCCI), Rohrbaugh et al. (50) described the discomfort program participants felt when members of the local community criticized Yale University's treatment of them as mere subjects for study (50). A structurally humble approach acknowledges the legitimacy of these perspectives and takes them into consideration when developing interventions. Montoya's third recommendation is for clinicians to be willing to admit that they do not know everything (i.e., “Be someone you'd like to know” (48, p. 153). This includes knowledge of oneself, not just one's implicit biases (51) but the various types of privileges that shape one's interactions with the world (52, 53). Finally, Montoya encourages clinicians to see their patients as more than just the problems they face (48). This means acknowledging the abilities that patients have in contributing to their care.

### Pedagogical Frameworks for a Structural Competency Curriculum

The proposed curriculum and its delivery draw on concepts from culturally relevant pedagogy (CRP) (54) and adult learning theory (55). Ladson-Billings developed CRP as an approach that draws on the cultural diversity of learners as a strength in the learning process, which helps to build “academic success,” “cultural competence,” and “sociopolitical consciousness” [(56, 57)—p. 75]. While generally grounded in a formal educational setting, the theoretical underpinnings of CRP are relevant in the clinical encounter since a critical role of clinicians is to provide patient education. When delivered in the context of a hierarchical relationship, such education is seldom effective because, in a structural competency framework, health is about more than individual behavior. When health care providers are made aware of the power differentials within the patient-provider relationship, they can approach patient education as an opportunity for mutual learning. Recognizing and incorporating the patient's lived experience in their delivery of services raises the social consciousness of health care providers beyond the immediate encounter.

As described in detail in the sections that follow, the approach to instruction presented here challenges practicing clinicians across the six domains of the andragogical framework (55). First, through didactic instruction, providers will gain a greater understanding of the importance of addressing the structural barriers their patients face to achieving optimal health outcomes. The content of this didactic training was recently delivered to an interdisciplinary group of graduate students participating in a Health Resources and Services Administration-funded program on the management of OUD in primary care (see Presentation 1 in Supplementary Material). Second, because the majority of practicing clinicians are unfamiliar with the concept of structural competency, the curriculum will encourage them to re-assess their awareness of the challenges their patients face. Third, by drawing on commonly encountered clinical challenges, providers will be able to contrast their own experiences with more comprehensive approaches to patient care. Fourth, the use of case studies will provide an opportunity to reflect on their readiness to manage the care of patients affected by the syndemic. Fifth, a structurally competent approach necessarily requires health care providers to re-orient their approach to care within broader societal structures. Finally, practice with the administration of structural vulnerability assessments will offer learners the opportunity to examine the quality of the questions they pose within the clinical encounter.

## The Learning Environment and Educational Format

A recent body of work has described structural competency training programs within medical schools, including training for pre-health/pre-med students, medical residents, and students of psychiatry (42, 43, 52, 58, 59). As described in Hansen and Metzl's (59) compendium of case studies, these efforts represent a small, but growing, number of interdisciplinary programs designed to bring awareness of structural influences on health into formal medical training programs (59). However, what is lacking is a training program that can address the knowledge and skills gaps of practicing providers. In the time-pressured environment of primary care, providers are unlikely to be willing to take the time to participate in tours of their surrounding communities to better understand the structural factors contributing to the challenges their patient's face in managing their health. However, it is critical to increase awareness of structural competency as a way to combat stigma in the syndemic and develop a more integrated approach to the provision of preventive and treatment services.

The goal of this section of the paper is to apply Metzel and Hansen's (1) five-part framework for improved structural competency in the design of a targeted educational curriculum on the syndemic that can be delivered on-site to practicing PCPs and their staff members (1). The proposed curriculum (Table 1) includes didactic lectures, interactive activities, case studies, discussions, individual practice assessment, and brainstorming. Altogether, the training is designed to take one and a half hours (i.e., 15 min to cover components 1 through 3 and 5 and 30 min to identify practice-specific interventions). Ideally, the training session will be followed with 3–6 monthly consultations to assist practices to implement workflow changes, applications, and other changes identified in the interventions phase. The objective is to test the feasibility of the program in a small sample (i.e., 3–5 practicing PCPs) and develop appropriate metrics to evaluate the model and refine it for further testing.

**Table 1:** Structural competency curriculum for addressing the syndemic in primary care.

**Module number/topic**	**Time (mins.)**	**Activities**	**Mode(s) of instruction**
1. Recognizing structural vulnerability in the syndemic	15	Didactic presentation - topics:^a^ • What's a syndemic? • Pharmaceutical companies' role in the opioid epidemic ◦ Marketing of opioid medications ◦ Understatement of opioid addictive potential • The War on Drugs and mass incarceration • The Ryan White HIV/AIDS Program • Federal funding priorities and HIV/HCV • Stigma's role in the syndemic	• Presentation • Group discussion
2. Taking a multidisciplinary approach to structural vulnerability	15	Discussion of tools for assessing vulnerability: • Social isolation as a risk factor in the syndemic ◦ UCLA Loneliness Scale (60) • Defining structural vulnerability ◦ Structural Vulnerability assessment (61)	• Self-assessment • Group discussion
3. Structural explanations in case studies	15	Case Studies in Social Medicine - from *The New England Journal of Medicine*: • “The Structural Violence of Hyperincarceration — A 44-Year-Old Man with Back Pain” (62) • “Structural Iatrogenesis—A 43-Year-Old Man with ‘Opioid Misuse”' (63)	• Group discussion
4. Structural Interventions	30	Practice assessment and brainstorming: • Review of tools for HIV/HCV/OUD screening • Process for applying for a DATA 2000 waiver • Using the PDMP for medication management • Identification of referral sources practice is currently lacking • Review of patient education relating to safe use, storage, and disposal of opioid medications	• Small group brainstorming • General discussion
5. Structural humility	10	Identity Wheel exercise (53)	• Self-assessment • Group discussion
6. Wrap up and next steps	5	Identification of additional resources/training needed for individual providers and the practice as a whole - possible examples: • Screening and Brief Intervention (SBI) for OUD (13) • Clinical protocols for management of HIV/HCV/OUD • Contingency management in the syndemic	• Individual practiceassessment • Brainstorming • Group discussion

a *See Presentation 1 in Supplementary Material*.

### Recognizing Structural Vulnerability in the Syndemic

The first phase of the proposed curriculum involves a didactic presentation that explores the constructs of structural competency and contrasts them with those of cultural competency and the social determinants of health (see Presentation 1 in Supplementary Materials). During this time, we will also provide an overview of the historical and social precedents (particularly stigma) that created the siloed systems of care for OUD, HIV, and HCV. This review will start by defining the syndemic and describing the interactions between the three conditions, as well as risks imposed by co-occurring substance use disorders. The presentation will include discussions of the role of pharmaceutical companies in marketing opioid medications to prescribers and minimizing the addictive potential of these drugs, as well as the racial implications of the “War on Drugs” and mass incarceration (26, 59, 64). It will provide a brief history of the Ryan White HIV/AIDS Program and the failure of the federal government to prioritize funding for the development of pharmaceutical treatments for HIV and HCV (65, 66). Finally, this segment of the curriculum will review Link and Phelan's model of stigma (10) and identify how integration of care for the syndemic within primary care practices can help to better integrate services and reduce syndemic-related stigma.

### Taking a Multidisciplinary Approach

Recent studies suggest that loneliness is prevalent throughout U.S. society (67–69). A study that used the University of California—Los Angeles's Loneliness Scale found that 46% of Americans report feeling alone at least some of the time (70). The study further indicated that, rather than alleviate the sense of loneliness, heavy use of social media is associated with a greater sense of loneliness (i.e., 73% of heavy social media users reported feeling alone vs. 52% among light users). During the second portion of the training, participants will review the Loneliness Scale (60) and will discuss the implications of loneliness on coping patterns (e.g., loneliness as an etiological factor contributing to substance misuse as a coping mechanism) and social engagement.

During this portion of the training, we will also present the Structural Vulnerability Assessment Tool developed by Bourgois et al. (61). The tool includes questions related to 8 structural dimensions (e.g., financial security, residence, risk environments, etc.), along with specific follow-up questions for each. For example, the question relating to residence asks, “Do you have a safe, stable place to sleep and store your possessions?” Follow-up questions include “How long have you lived/stayed there? Is the place where you live/stay clean/private/quiet/protected by a lease?” (68, p. 15) We will review the tool and talk about the practice's readiness to incorporate the items in health assessments.

### Identifying Structural Explanations for Health Outcomes in the Syndemic

In its Perspective section, the *New England Journal of Medicine* has a regular feature called “Case Studies in Social Medicine.” These articles highlight real cases and examine the structural implications inherent in patients' interactions with the health care system. During this section of the training, participants will review up to two cases relating to patients with complaints of chronic pain and examine the traditional approach they would take to these cases vs. one that considers structural factors. One goal will be to discuss how structural factors are frequently seen as cultural representations. The emphasis will be on understanding how to break down stereotypes to identify the structural forces that create risks and barriers that cross ethnic and socioeconomic lines.

### Implementing Structural Interventions in Primary Care

Because the goal of the training is to motivate providers to implement changes in their practice, the training will include 30 min to discuss specific structural interventions that practices can implement to address the syndemic. Specifically, we will first assess the extent to which practices are engaging in routine HIV, HCV, and OUD screening according to guidelines; implementing harm reduction interventions (e.g., prescription of PrEP and naloxone distribution); employing prescribers with DATA 2000 waivers to prescribe buprenorphine for OUD; and using the State PDMP for prescription opioid management, particularly among patients with infectious diseases. To the extent that practices do not have these systems in place, or are not using them efficiently, we will provide information, training, scripts, and tools to facilitate their uptake. Based on the prior discussions, we will also review the practice's list of referral sites and identify gaps in services for which new sites of referrals can be developed. Finally, we will review the practice's educational initiatives relating to the safe use, storage, and disposal of opioid medications and will ensure that sites have a list of local disposal sites of opioid medications and information that they can provide to patients regarding when and how to dispose of medications safely when there are no local drop-off sites available. The goal will be to help practices develop a more integrated system of care that can overcome clinical inertia for managing the syndemic.

### Approaching the Syndemic With Humility

The last portion of the training will focus on recognizing privilege as a component of structural humility. We will use an abbreviated version of the Identity Wheel exercise described by Chow et al. (53). The activity involves participants filling out two rings of a circle, one which includes given identities (e.g., age, nationality, language) and the other that includes chosen identities. After participants fill out their wheels, they engage in directed discussions regarding the meanings of their social identities. The goal is for participants to understand that there are identities that are salient to others that are not as relevant to their own experiences. Under the original model, the activity is expected to last at least 40 min. Due to time constraints, the activity for this training will focus on a shortened list of discussion questions, specifically, those focusing on identities that privilege providers in their professional roles and how these experiences differ from those of their patients.

### Program Wrap Up

The final portion of the program will focus on lessons learned and next steps. As prior researchers have noted, there are many successful models of primary care, office-based management for intersecting disorders (13, 33). However, approaching care within the syndemic requires an individualized approach that addresses the strengths and needs of the specific clinical practice site (33). As such, the final 5 min of the training will involve a summary of lessons learned and identification of additional training that individual clinicians may need, or workflow processes that need to be revised at the practice level.

### Approach to Assessment

Assessment of participant learning will be based on Bloom's Taxonomy (71). Specifically, we will focus on the knowledge, skills, and attitude domains. Specially, for the knowledge domain, we will assess participants' ability to apply concepts of structural competency in their discussions of the case studies. Through this activity, participants will be able to demonstrate their ability to evaluate their current practice and conceptualize approaches that are more responsive to the structural challenges their patients face. In the domain of skills, participants will have the opportunity to practice data gathering using the Structural Vulnerability Assessment Tool (61) and to adapt the tool to the needs of their practice. Finally, through a post-course evaluation, participants will have the opportunity to reflect on the learning and share their perceptions regarding the utility of the structural competency approach and their intentions to implement changes in their practice.

## Discussion

Primary care practices are ideal settings for addressing the syndemic. Evidence shows that many people who inject drugs or are at risk for infectious diseases see their PCPs on a yearly basis but are not engaged in discussions about harm reduction; in many cases, the PCP is not even aware of the patient's risk status (72). Normalizing the management of OUD, HIV, and HCV in the primary care setting can help to reduce the stigma that exacerbates poor health outcomes in the syndemic (13, 21, 73). Until the barriers (including lack of awareness or clinical inertia to prescribe buprenorphine and federal regulations restricting methadone outside of opioid treatment programs) are removed, PCPs should be encouraged to complete training to prescribe buprenorphine and train patients in overdose prevention with naloxone (14, 21, 74). As treatment regimens have become more efficacious and simpler, PCPs should be encouraged to accept the responsibility for medical management of patients with substance use disorder, HIV, and HCV (14, 75). Training in structural competency will help these providers understand that the conditions underlying these intersecting disorders (e.g., stigma, social isolation, and disadvantage) are ideally addressed in settings that promote frequent contact and enhanced trust (13, 14, 26, 32). The goals of the proposed curriculum are 2-fold. First, we seek to expand existing models of structural competency training to target other disciplines, particularly PCPs in active practice. Second, while the proposed training focuses on the theoretical and practical aspects of the syndemic, it also incorporates practical, hands-on activities that can be readily implemented in the busy primary care setting.

The ultimate objective is to deploy and evaluate the training within a sample of primary care practice sites across New Jersey. We expect this to be a multiphase process. The preliminary phase will be a feasibility trial with three to five practicing PCPs to test the content and timing of the various activities. Information gleaned from this trial will be used to refine the content and identify appropriate clinical markers of program efficacy. Obvious objective candidate measures include stigma reduction; number of patients screened for HIV, HCV, and OUD; number of new applications for DATA 2000 waivers; and number of times the PDMP is consulted when prescribing opioid medications. More subjective indicators of program success would include satisfaction with the program and confidence in assessing patients for structural barriers. The long-term goal is to implement the training practice-wide to enhance the structural competency of the entire clinical setting. Eventually, we plan to apply for continuing education credits to implement and test the program across a wide range of practice settings.

## Data Availability Statement

The original contributions presented in the study are included in the article/Supplementary Materials, further inquiries can be directed to the corresponding authors.

## Author Contributions

The author confirms that all work conducted in the development of this paper was solely by AB.

## Conflict of Interest

The author declares that the development of this paper was conducted in the absence of any commercial or financial relationships that could be construed as a potential conflict of interest.
