# Supplementary material for: A Structural Competency Curriculum for Primary Care Providers to Address the Opioid Use Disorder, HIV, and Hepatitis C Syndemic
Source: Front Public Health. 2020 Jun 5;8:210. doi: 10.3389/fpubh.2020.00210 (PMC7289946; doi:10.3389/fpubh.2020.00210)
Supplement: Supplementary file 1 [file Presentation_1.pdf]

Developing Structural Competency to Address the Opioid Use Disorder, HIV, and HCV Syndemic in Primary Care

Syndemic

Noun /sin″dem′ik/ (*plural* syndemics)

- "Two or more epidemics (i.e., notable increases in the rate of specific diseases in a population), interacting synergistically and contributing, as a result of their interaction, to excess burden of disease in a population" (Singer & Clair, 2003, p. 425)

The Opioid, HIV, Hepatitis C Syndemic

- 47,600 Americans died of opioid overdoses in 2017 (CDC, 2018)
- Prevalence of infectious disease among people who inject opioids
  - *Hepatitis C virus (HCV): 67% (Nelson et al., 2011)*
  - *HIV: 33% (Mathers et al., 2007)*
- 10% of people living with HIV are infected through injection drug use (IDU)
- 2015 Scott County Indiana HIV outbreak – 181 new HIV diagnoses (Peters et al., 2016)
  - *88% had injected extended-release oxymorphone*
  - *90% were coinfected with HCV*

Addressing the Syndemic

- The opioid/HIV/HCV syndemic has been described as one of the most pressing public health issues facing the United States today (Perlman and Jordan, 2018)
- Addressing it requires a collaborative and coordinated approach that
  - *Prevents and treats the individual components*
  - *Tackles the factors that create disease overlaps*
- Primary care providers must understand how stigma associated with the syndemic perpetuates illness and social inequality

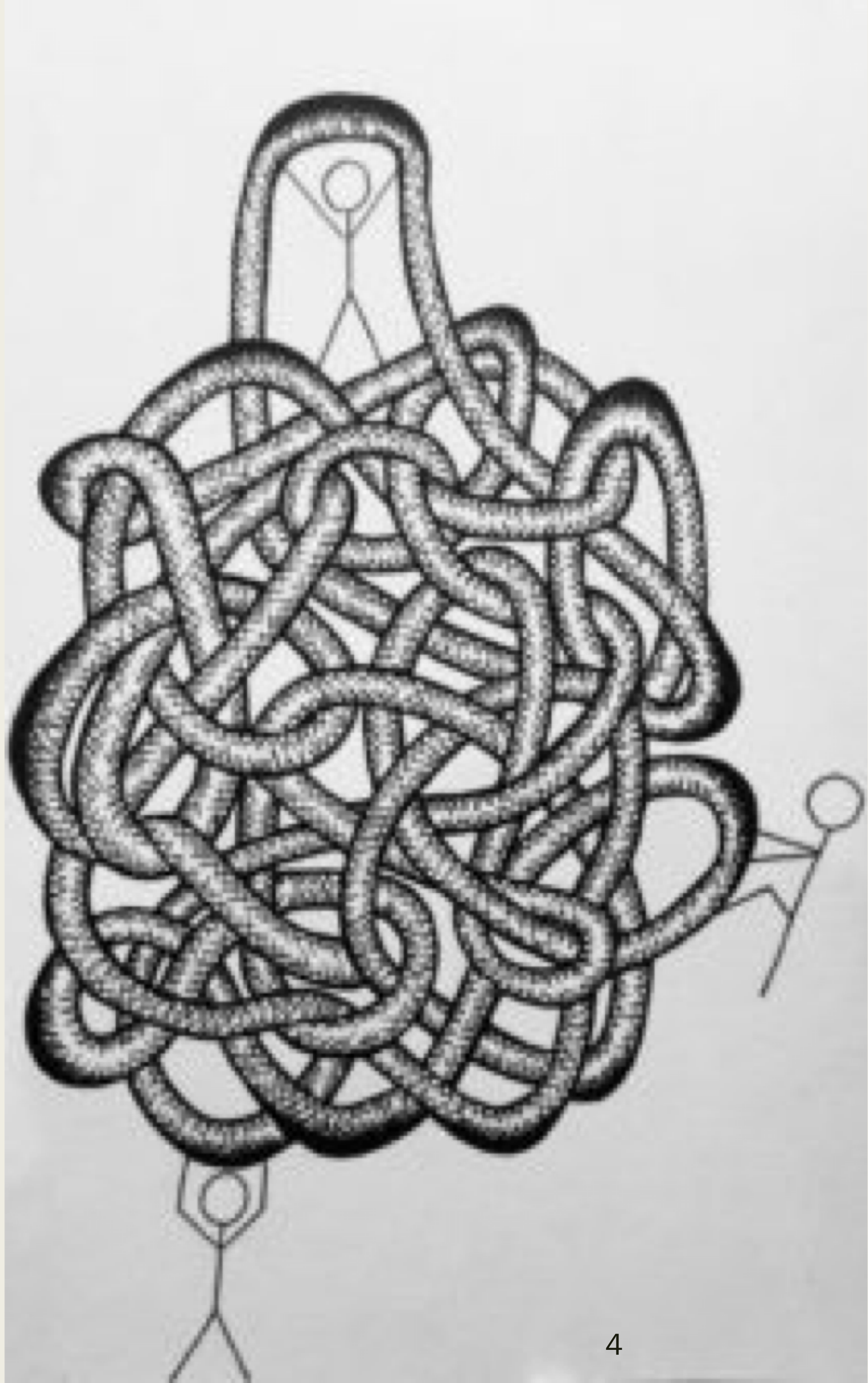

Stigma Framework (Link & Phelan, 2001)

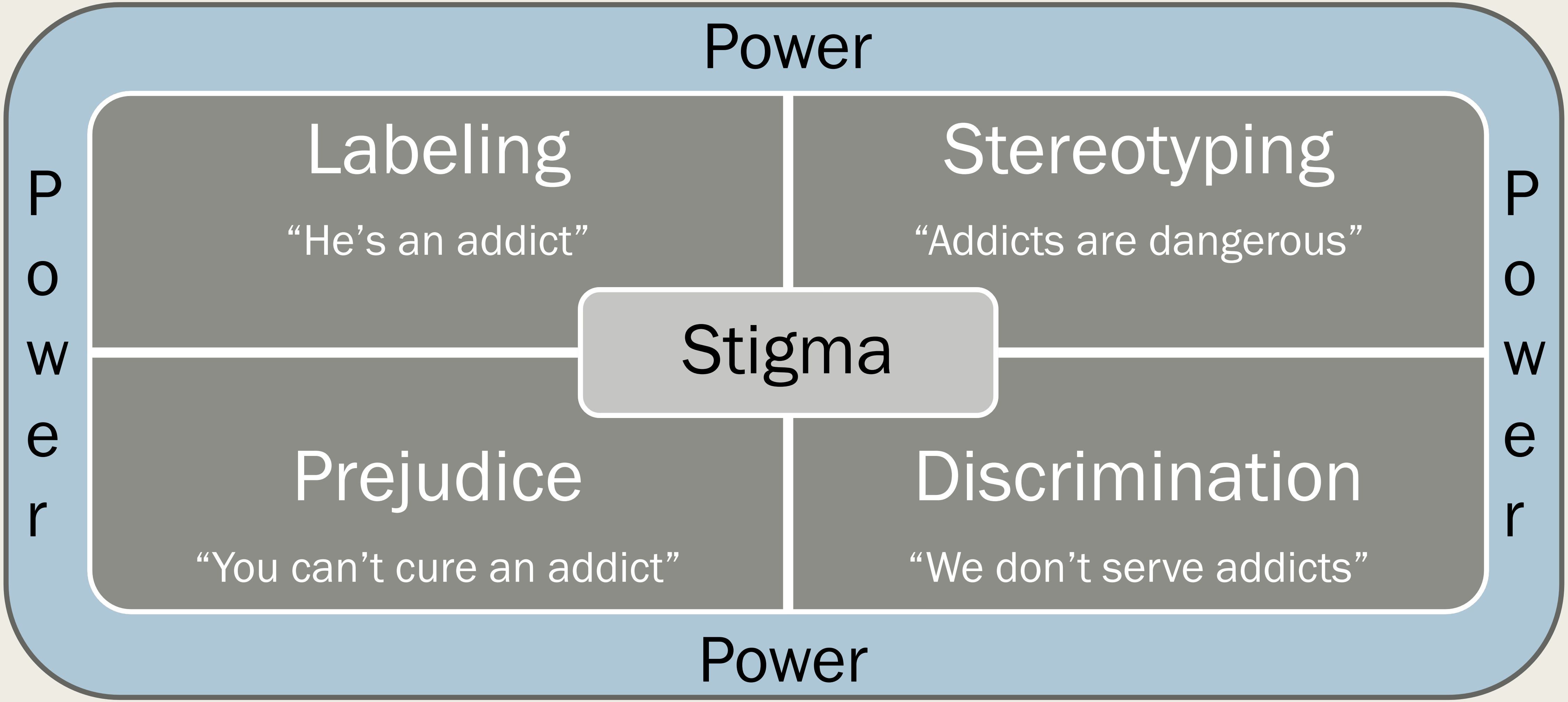

Stigma in the Syndemic

Seven Types of Stigma

Type 1	Type 2	Type 3	Type 4	Type 5	Type 6	Type 7
**Self Stigma**	**Perceived Stigma**	**Label Avoidance**	**Health Practitioner Stigma**	**Public Stigma**	**Stigma by Association**	**Structural Stigma**
Self-stigma happens when the person with substance use disorder internalizes negative attitudes.	Perceived stigma is the belief that others have negative beliefs about people with substance use disorder.	This is when a person chooses not to seek substance abuse services to avoid being assigned a stigmatized label.	This takes place any time a health professional allows stereotypes and prejudices about substance abuse to negatively affect a client's care.	Occurs when the public endorses negative stereotypes and prejudices, resulting in discrimination against people with substance use disorder.	This occurs when the effects of stigma are extended to someone linked to a person with substance use disorder.	Institutional policies or other societal structure that result in decreased opportunities for people with substance use disorder.
**Example**: "I don't deserve to see my kids until I get clean."	**Example:** "There's no use attending that event. No one wants to be around someone with substance abuse."	**Example:** "I don't want to go to rehab. They'll just say I'm a 'junkie'."	**Example:** "I'm not going to refill your medication because you'll just sell it to get high."	**Example:** "We don't allow people who abuse substances to participate in our program because they are dangerous."	**Example:** "I don't spend time with Linda anymore. Her son abuses heroin and I don't want people to think I condone that behavior."	**Example:** "Law prohibits your receipt of housing assistance if you are currently abusing prescription opioid medications."

(Adapted from Grappone, 2017)

Structural Vulnerability

- Confluence of structural barriers and social conditions that undermine health status and outcomes
- Vulnerability index – 15 indicators (Van Handel, et al., 2015)
  - *Reflects Census tracts with higher probabilities of infectious disease outbreaks among people who inject drugs*
  - *6 key constructs*
    - Overdose death rates
    - Rx opioid sales
    - Buprenorphine capacity via waiver
    - % non-Hispanic white ethnicity
    - Per capita income
    - Unemployment rate
  - *220 counties in the 95th percentile of vulnerability*

Structural Vulnerability

- Confluen[illegible] health status and outc[illegible]
- Vulnerabi[illegible]
  - *Refle[illegible] se outbreaks amo[illegible]*
  - *6 ke[illegible]*
    - Ov[illegible]
    - Rx[illegible]
    - Bu[illegible]
    - % [illegible]
    - Pe[illegible]
    - Ur[illegible]
  - *220[illegible]*

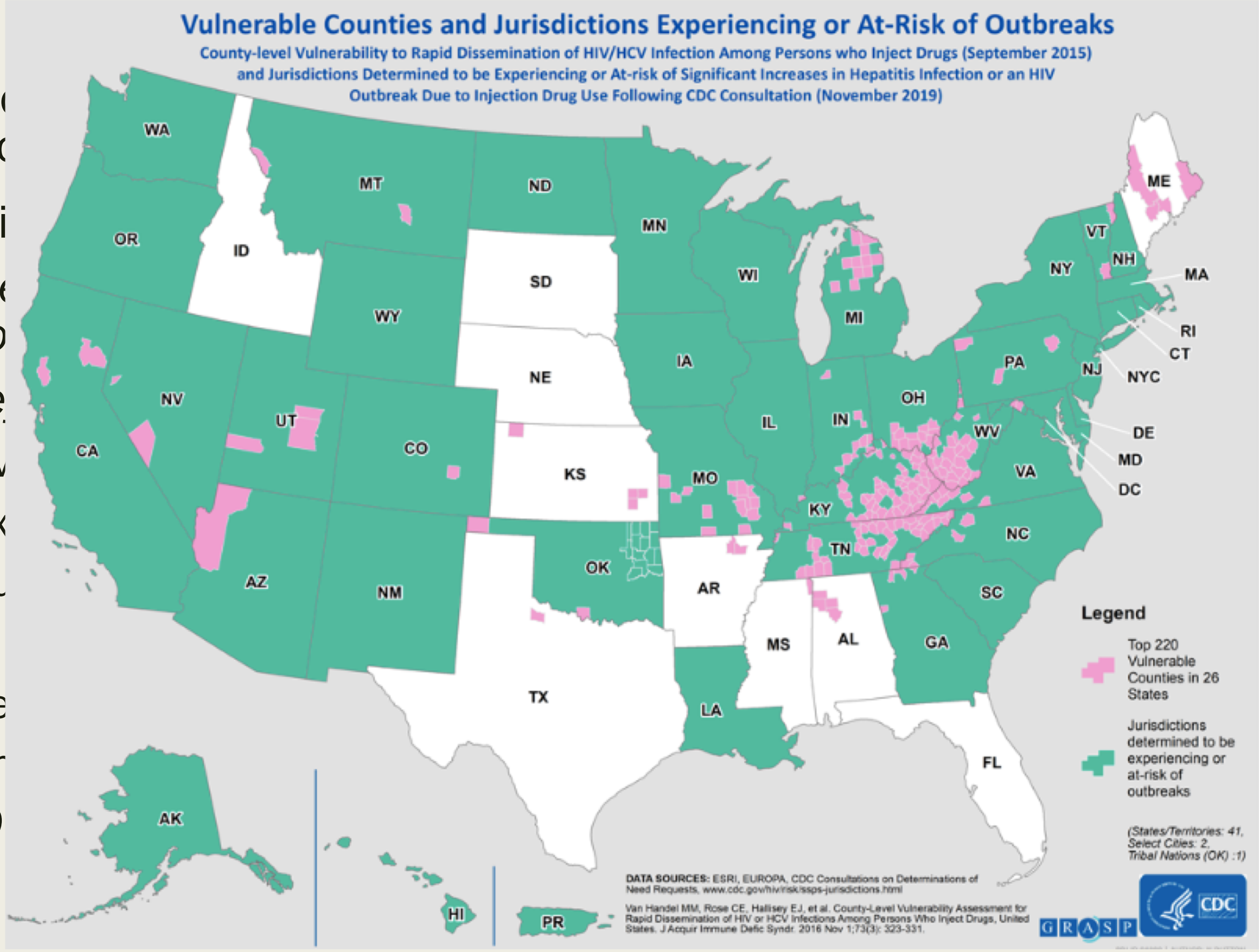

Structural Competency

- Practice of social reflection that promotes understanding of how upstream decisions perpetuate and promote downstream inequities
  - *Housing policies*
  - *Transportation infrastructure*
  - *Supply chains for food and health care delivery systems*
  - *Criminalization (e.g., vagrancy, HIV, poverty)*
  - *Medicalization of health care*
  - *Definitions of illness and health*
- Bridges social determinants of health and clinical interventions
- Supports an "enabling environment" for self-care interventions (World Health Organization, 2019)

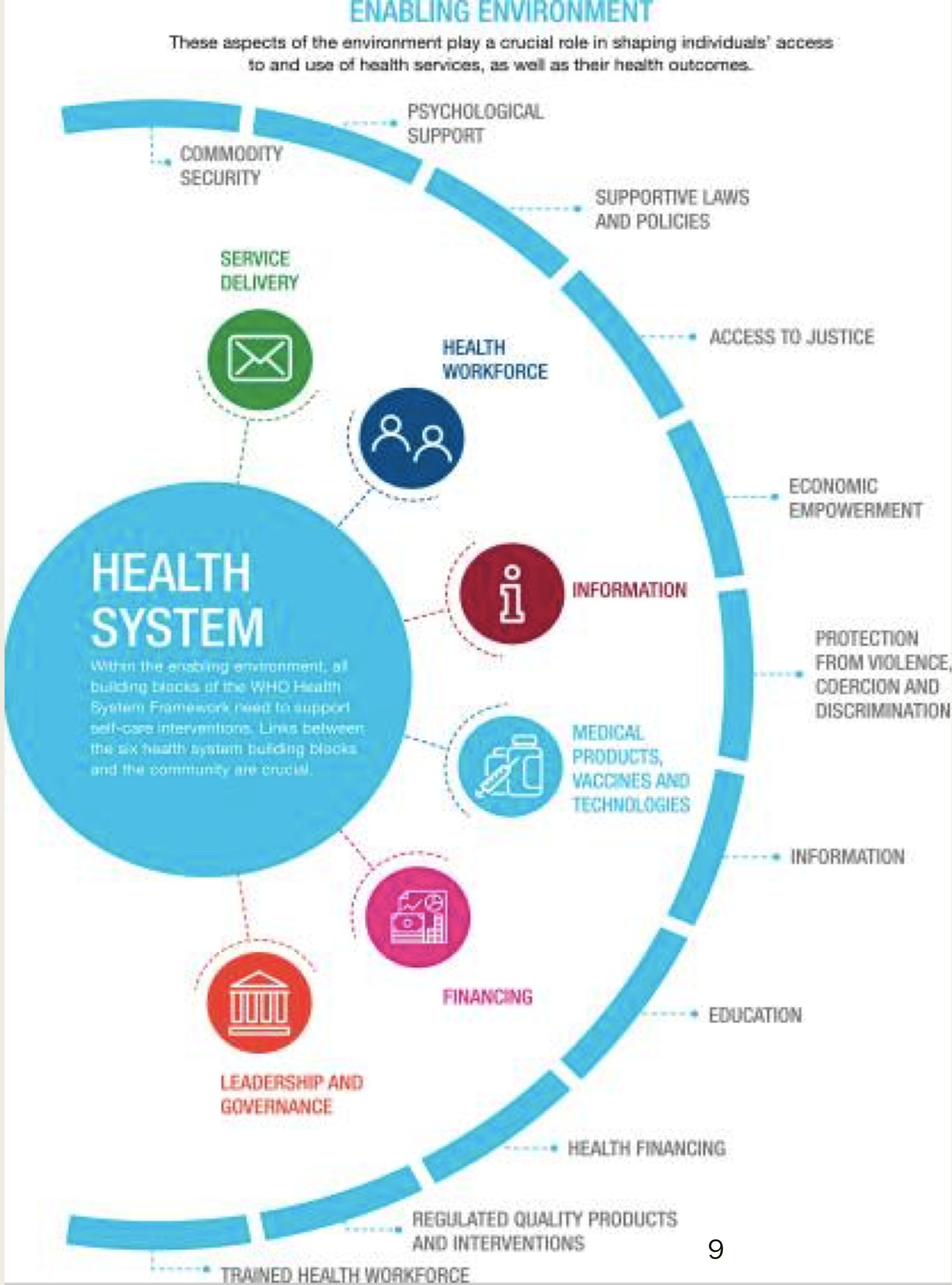

Cultural vs. Structural Competence

- Cultural Competency:
  - *Respect for cultural health beliefs*
  - *Elimination of language barriers*
  - *Addressing health literacy*
  - *Awareness of implicit bias*

  ➔ *Focus is on identifying clinician bias and improving communication*

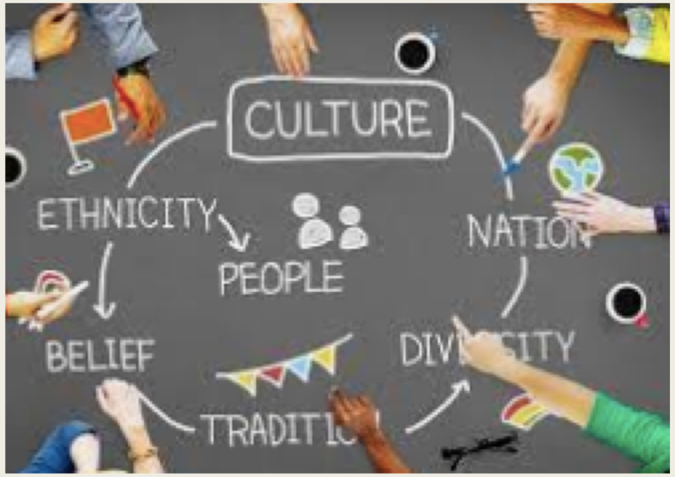

- Structural Competency:
  - *Recognition of how context affects symptom presentation*
  - *Clinicians expected to advocate for corrective actions*

  ➔ *Goal is to enhance recognition of social, economic, and political structures that lead to health inequities*

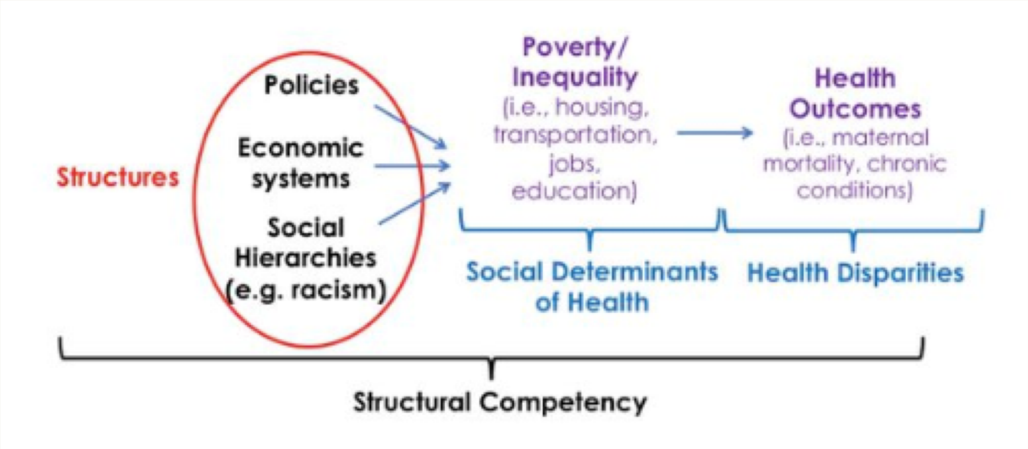

Equality, Equity, and Justice

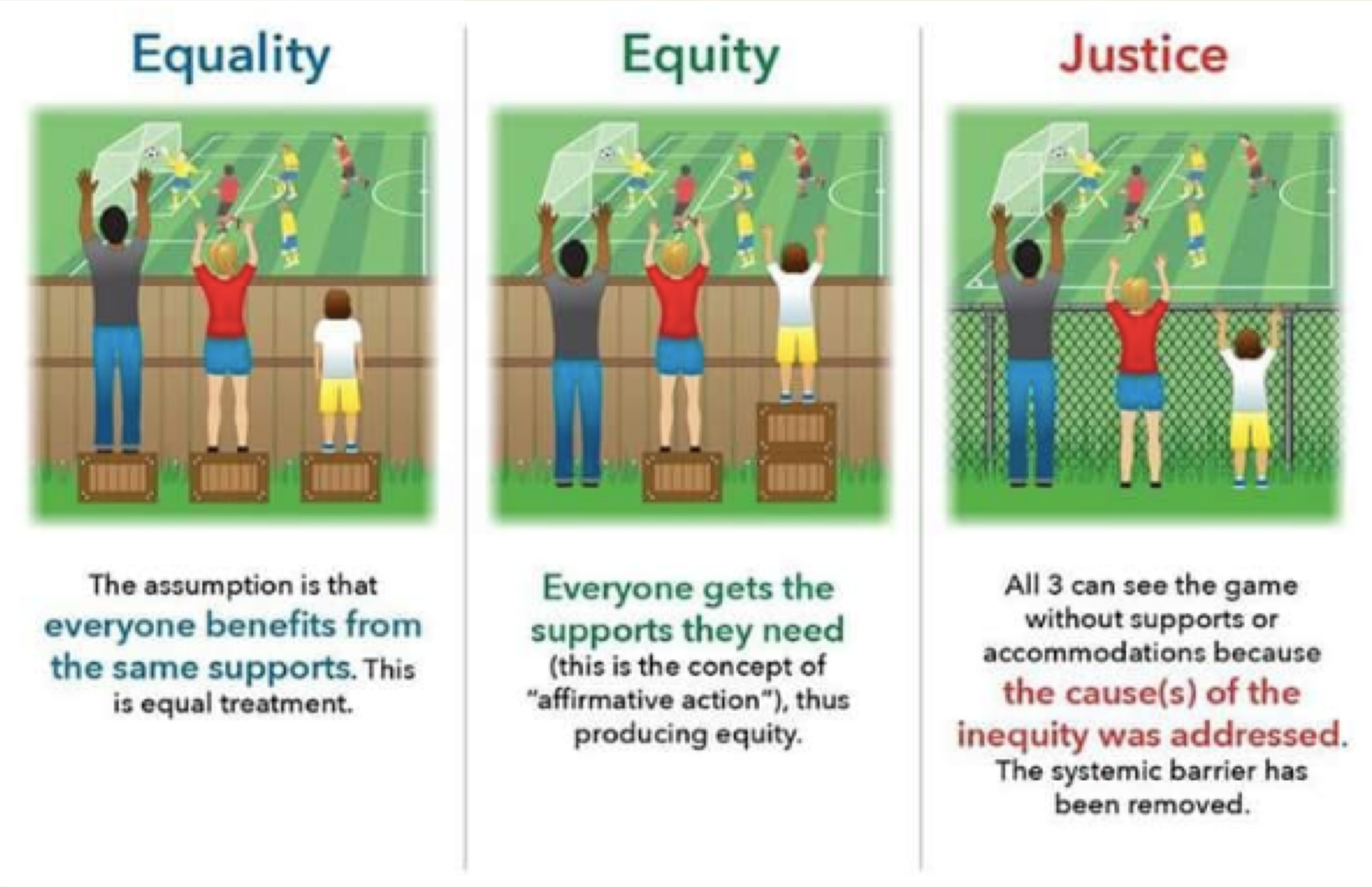

8 Structural Factors (Metzl & Hansen, 2014)

Access to Health Care

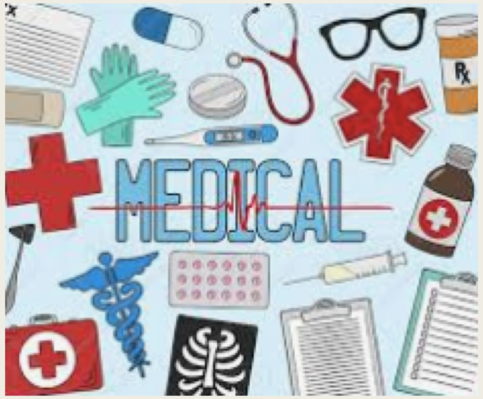

Health Insurance

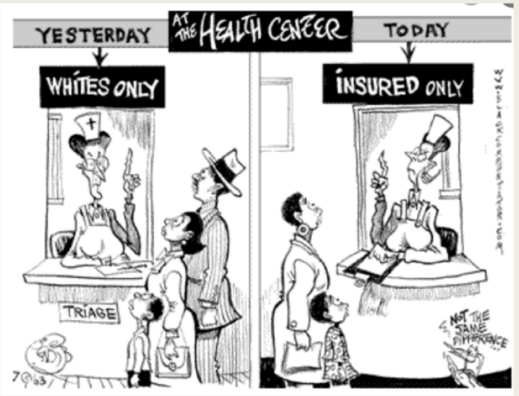

Medicalization

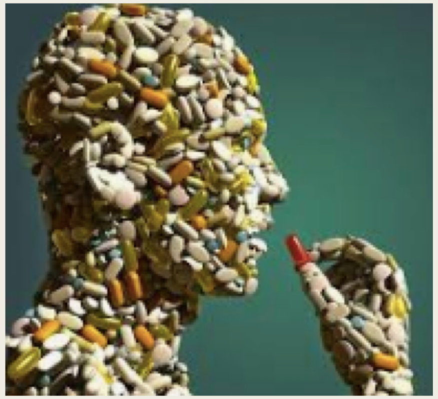

Neighborhood

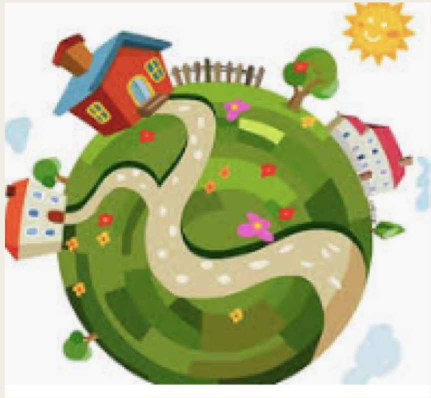

Health Care Delivery

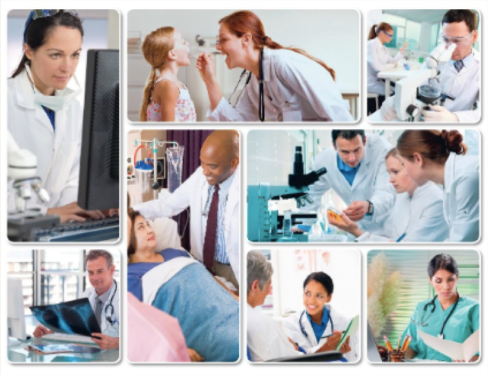

Institutional Racism

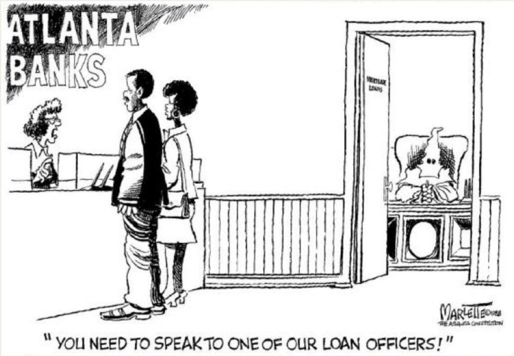

Household Income

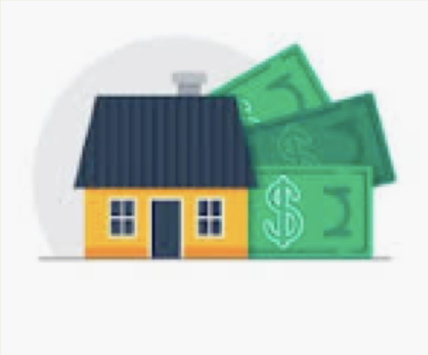

Social Policies

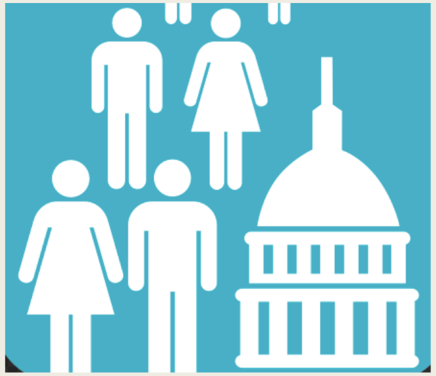

Metzel & Hansen’s (2014) Structural Competency Framework

Comprised of ”five intersecting skill-sets” (p. 6)

1) Recognizing the structures that shape clinical interactions
2) Developing extra-clinical language of structure
3) Rearticulating “cultural” presentations in structural terms
4) Observing and imagining structural interventions
5) Developing cultural humility

Clinical Scenario

"Mrs. Jones is an African American woman in her mid-60s who comes late to her office visit and refuses to take her blood pressure medications as prescribed."

- *Metzel & Hansen (2014, p. 2)*

1. Recognizing Structures that Shape Clinical Interactions

- Need to recognize how structural factors affect medical decision-making

> *"Mrs. Jones is an African American woman in her mid-60s who comes late to her office visit and refuses to take her blood pressure medications as prescribed."*

Traditional Approach:
- Socioeconomic background
- Cultural beliefs
- Health literacy
- Lifestyle factors
- Physician bias
- Trust of health care system
- Others?

Structurally Competent Approach:
- Time allotted to exam by insurance companies
- Limited funding for research on alternative therapies
- Biomedical perspective of the healthcare system
- Policies regarding the prices of prescription drugs
- Culture of diagnosis and pharmaceutical treatment
- Others?

2. Developing an Extra-clinical Language of Structure

- Understanding structure from a multidisciplinary (e.g., urban planning, sociology, economics, history, etc.) perspective

"Mrs. Jones is an African American woman in her mid-60s who comes late to her office visit and refuses to take her blood pressure medications as prescribed."

Traditional Approach:
- Social determinants of health
- Health disparities
- Policy changes
- Investments in impoverished areas
- Others?

Structurally Competent Approach:
- Social hierarchies
- Nature of racism
- Structural violence
- Political systems that promote stigma
- Systems of privilege
- Others?

3. Rearticulating "Cultural" Presentations in Structural Terms

- Identifying how structures create barriers to care and inequities

"Mrs. Jones is an African American woman in her mid-60s who comes late to her office visit and refuses to take her blood pressure medications as prescribed."

Traditional Approach:
- Familiarity with cultural values
- Ethnic identity
- Cultural value of timeliness
- Others?

Structurally Competent Approach:
- Local transportation infrastructure
- "Strategies" for getting to the clinic
- Biases in diagnosis that require frequent visits
- Institutional racism
- White privilege
- Others?

4. Observing and Imagining Structural Interventions

- Understanding that structures are human creations and are not immutable

"Mrs. Jones is an African American woman in her mid-60s who comes late to her office visit and refuses to take her blood pressure medications as prescribed."

Traditional Approach:
- Pill organizers
- Phone-based reminders
- Educational brochures
- Transportation vouchers
- Reminder phone calls
- Others?

Structurally Competent Approach:
- "Prescribing" food – bills sent to health center
- Eliminating medical debt
- Creating community spaces in urban areas
- Refrigerated trucks to deliver food to rural areas
- Patient-defined goals
- Community-based participatory research
- Others?

5. Developing Structural Humility

- Remaining open to the evolving nature of a patient's narrative

"Mrs. Jones is an African American woman in her mid-60s who comes late to her office visit and refuses to take her blood pressure medications as prescribed."

Traditional Approach:
- Patient chart as a definitive history
- Medical authority
- Static referrals to specialty care
- Focus on biomedical goals
- Others?

Structurally Competent Approach:
- Ongoing dialogue to identify new challenges
- Developing new networks to meet patient needs
- Patient as co-equal collaborator/authority
- Recognizing limitations of medical expertise
- Others?

Example Training Programs

- Albert Einstein College of Medicine
  - *Research-base health activism program*
  - *Physicians as advocates for "public health, social justice, and health equality"*
- University of Michigan - Critical Consciousness (Kumagai & Lypson, 2009)
  - *Understanding medicine in the context of society, culture, and history*
  - *Identifies, and seeks to address, social problems*
- Accreditation Council for Graduate Medical Education
  - *Health disparities competencies*
  - *Quality Improvement: Health Care Disparities Collaborative*

Goals for Structural Competency

- Ongoing engagement and learning about structural determinants
- Multidisciplinary engagement beyond health-related disciplines
- Recognition of the possibility for personal growth and practice
- Developing habits and practices that promote active engagement among other providers
- Active involvement in social, political, and economic activities to promote health

Applying Metzel & Hansen's Framework to the Opioid/HIV/HCV Syndemic

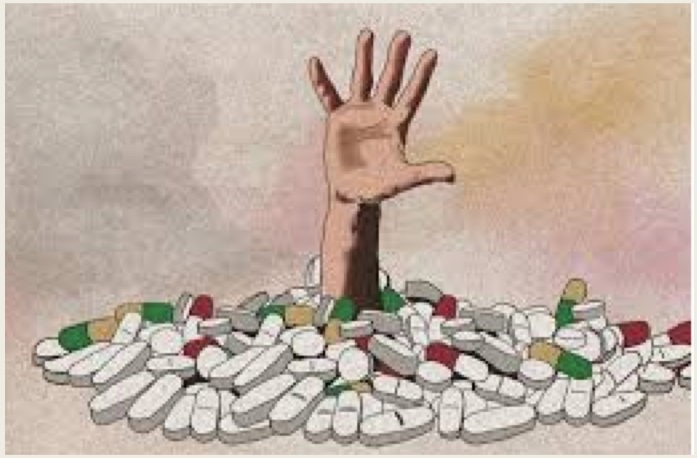

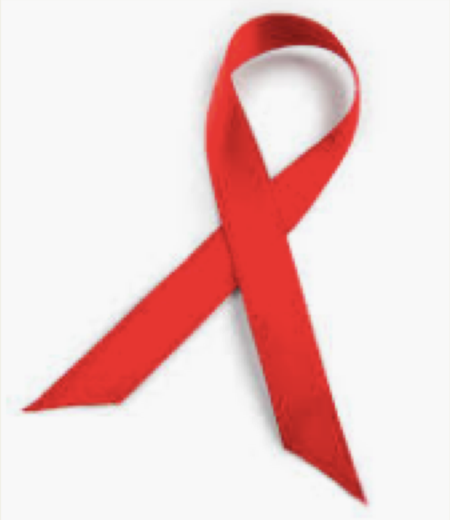

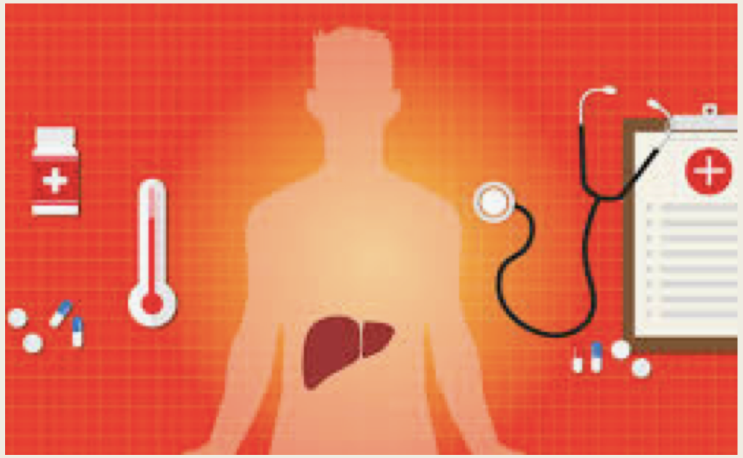

1. Recognizing Structures that Shape Clinical Interactions

- Opioids
  - *Marketing of prescription opioid medications (e.g., pain as the 6th vital sign)*
  - *Targeting areas with existing pain management clinics*
  - *Pharmaceutical companies understating the addictiveness of opioids*
- HIV
  - *Development of separate systems of care for people affected by HIV*
  - *Federal government's lack of investment in drug development*
- HCV
  - *High cost of effective treatments*
  - *Insurers restricting access (e.g., excluding people who use drugs)*

2. Developing an Extra-clinical Language of Structure

- Social Networks: "Loneliness is at Epidemic Levels in America" (Cigna, 2020)
  - *The role of social media (73% of heavy users vs. 52% among light users)*
  - *Screening tools for Depression, Alcohol and Drug Use, and Loneliness*
  - *Reimbursement for preventive care services*
- Infrastructure: Inadequate transportation as a barrier to healthcare engagement
- Financial services: housing instability
  - *Prejudged: The Stigma of Eviction Records*
  - *Kept Out: lenders perpetuate ethnic wealth gaps*
- Legal system: the criminalization of drug use

Structural Stigma and Discrimination: Examples

- Quiz: Could You Manage as a Poor American? (New York Times, January 28, 2020)
- Institutional and Structural Barriers to HIV Testing (Meyerson et al., 2014)
  - *Institutional discrimination (intentional acts)*
    - Staff reacting negatively to request for an HIV test
    - Staff expressing judgments about patients' presumed HIV risk behaviors
  - *Structural discrimination (unintentional)*
    - Separate systems of care for people living with/at risk for HIV
    - Limiting HIV testing to Wednesdays only
    - Test site difficult to find (in basement)
    - "Public" waiting room mixed with WIC recipients (forces disclosure)
- Policies restricting voting rights

Structural Stigma and Discrimination: Exa

- Quiz:
  28, 2020)
- Instit
)
  -
    rs
  -
- Polici

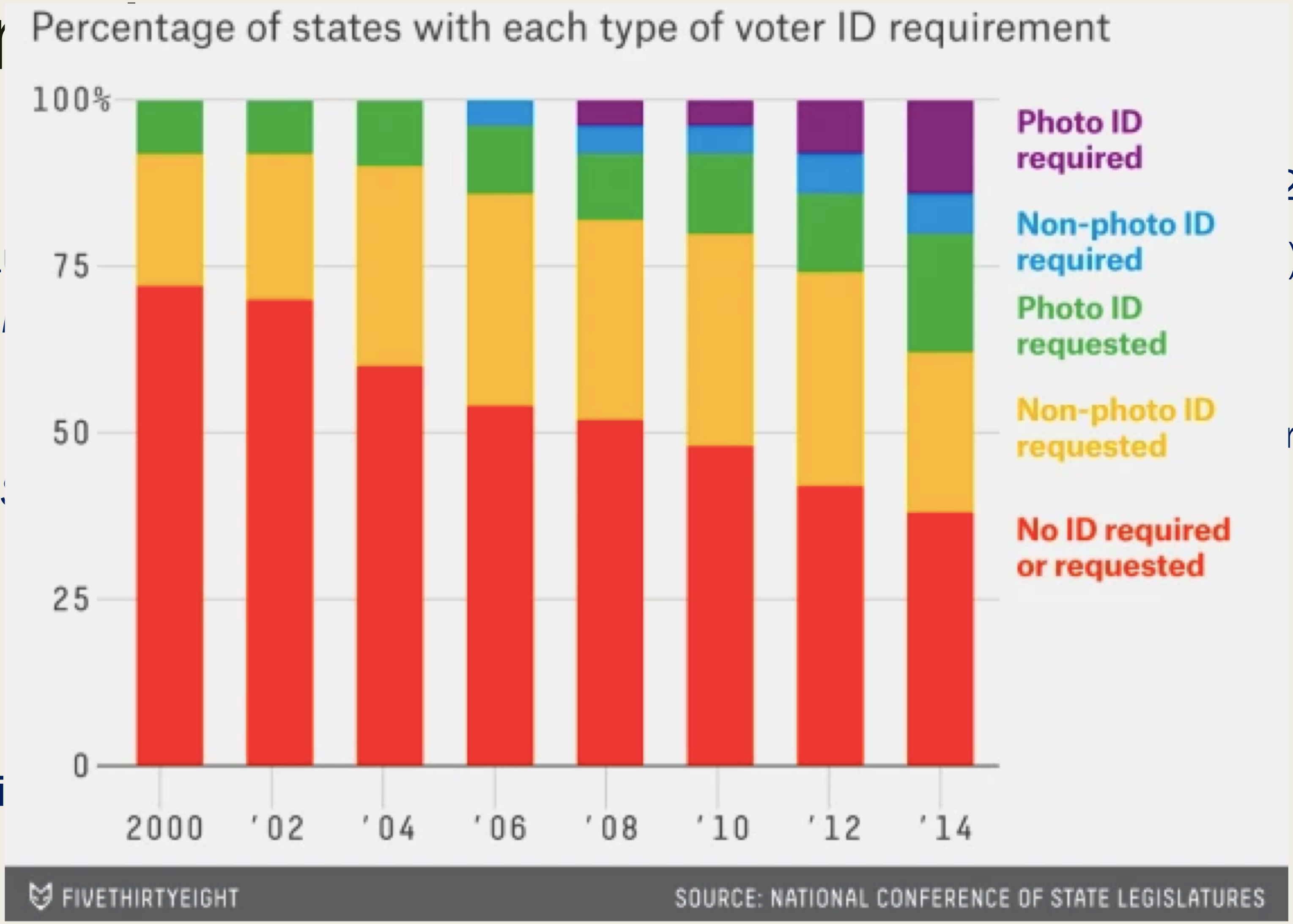

Structural Stigma and Discrimination: Examples

- Quiz: Could You Manage as a Poor American? (New York Times, January 28, 2020)
- Institutional and St [illegible] et al., 2014)
  - *Institutional di* [illegible]
    - Staff reacti [illegible]
    - Staff expres [illegible] risk behaviors
  - *Structural disc* [illegible]
    - Separate sy [illegible] HIV
    - Limiting HI [illegible]
    - Test site difficult to find (in basement)
    - “Public” waiting room mixed with WIC recipients (forces disclosure)
- Policies restricting voting rights

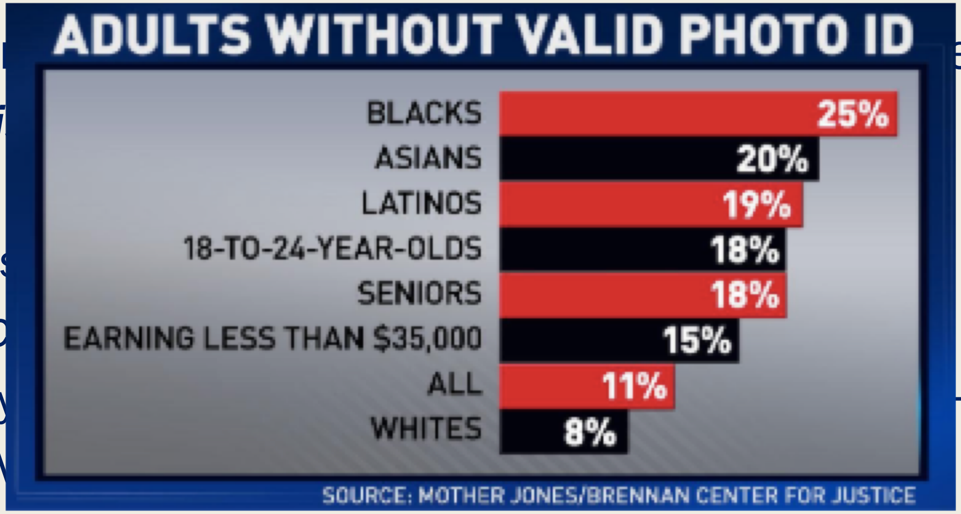

Structural Stigma and Discrimination: Examples

How Gerrymandering Works

- Quiz: [illegible] 2020)
- Instit[illegible]
  - [illegible]
  - [illegible]
- Polici[illegible]

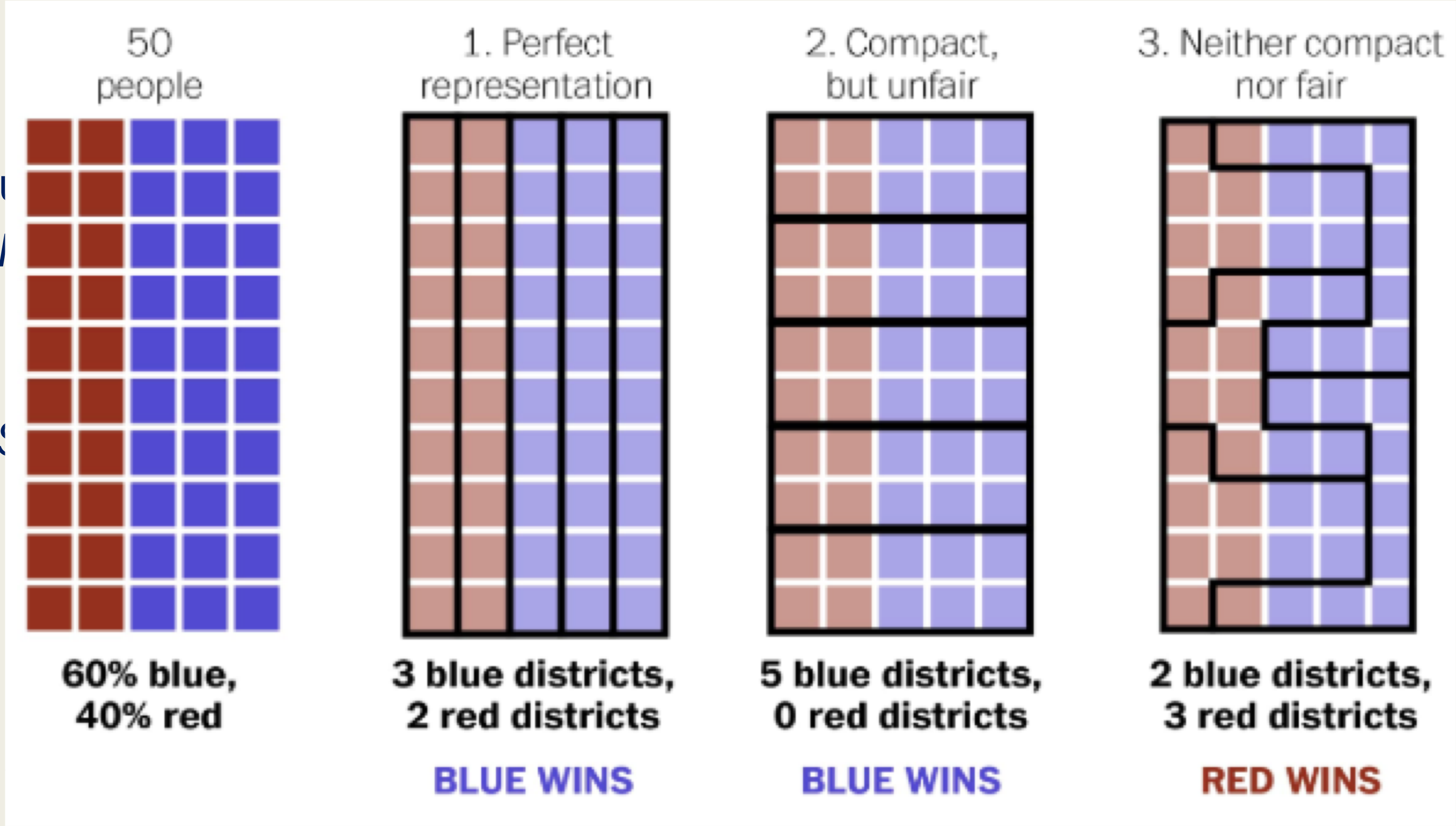

Structural Stigma and Discrimination: Examples

Gerrymandering in North Carolina

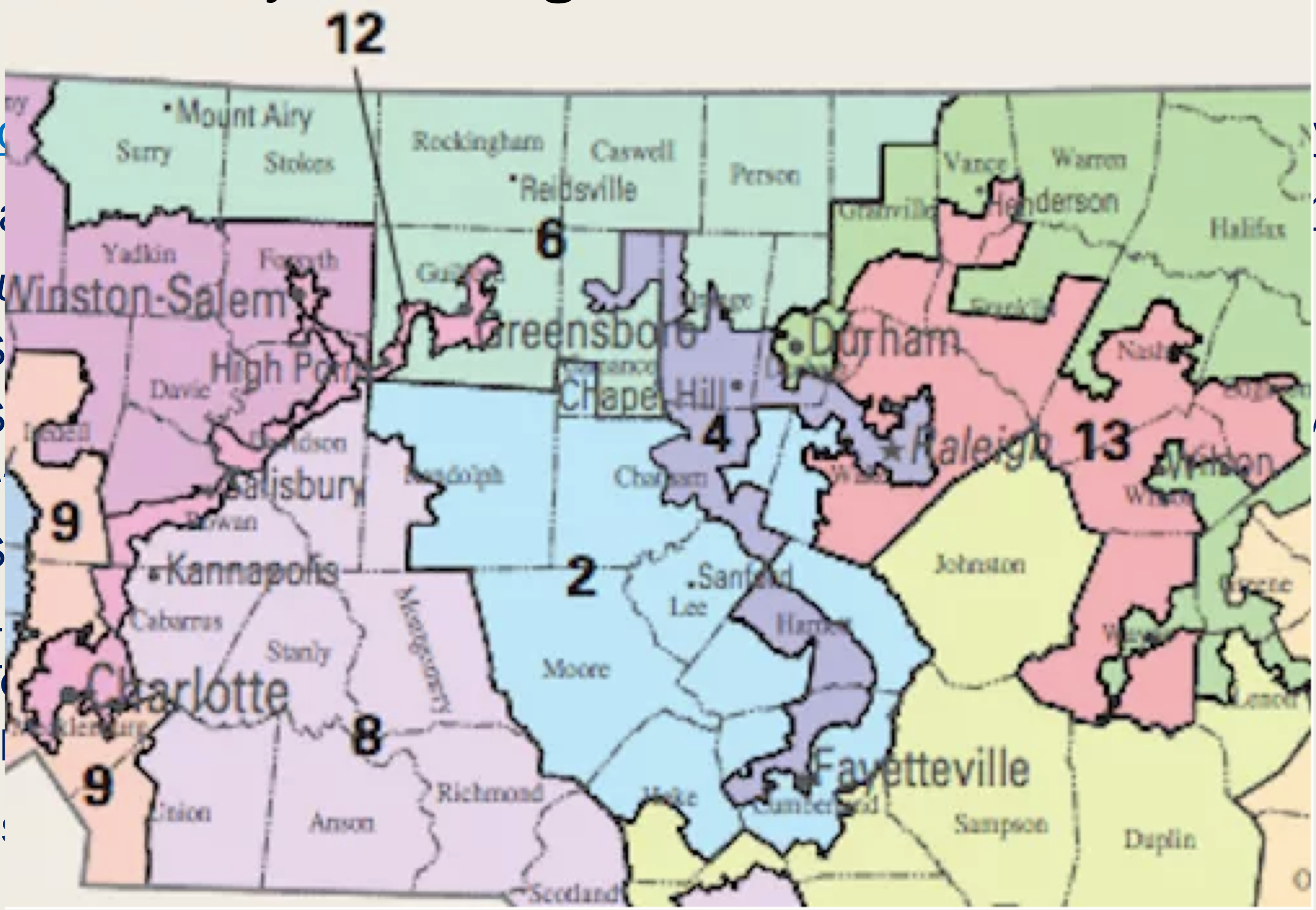

- Quiz: Coul[illegible] y 28, 2020)
- Institution[illegible] 14)
  - *Institu*[illegible]
    - S[illegible]
    - S[illegible] viors
  - *Struc*[illegible]
    - S[illegible]
    - L[illegible]
    - T[illegible]
    - “[illegible]
- Policies res[illegible]

3. Rearticulating "Cultural" Presentations in Structural Terms

- Higher prevalence of cardiovascular disease:
  - *Cultural: due to culture-based dietary choices*
  - *Structural: living in low-resource areas (e.g., food deserts, life expectancy by zip code)*
- Mental illness:
  - *Cultural: genetic predisposition*
  - *Structural: poverty affects brain development*
- Late diagnosis of HIV infection (i.e., AIDS diagnosis):
  - *Cultural: lack of trust in the health care system*
  - *Structural: Medicaid programs do not cover HIV prevention/care until an AIDS diagnosis*

4. Observing and Imagining Structural Interventions

- Providing OUD, HCV, and HIV screening according to evidence-based guidelines
- Increasing access to medication-assisted treatment (MAT) for OUD
  - *Expanding number of primary care providers with Drug Addiction Treatment Act of 2000 (DATA 2000) waivers to prescribe buprenorphine*
  - *Increased funding for MAT treatment in primary care settings*
- Expanding needle exchange programs and safe injection sites
- Removing insurance barriers to evidence-based treatments
- Enhancing referral networks to specialty services within primary care settings
- Active promotion of social justice in health care

5. Developing Structural Humility

- Taking a patient-centered approach
  - *Being open to patients' perspectives, even if they judge us in a negative light*
- Understanding the structure of privilege
  - *Ethnicity: What is White Privilege? (Tim Wise)*
  - *Physical abilities: Able-Bodied Privilege Checklist*
  - *Gender: 160+ Examples of Male Privilege*
  - *General: How Privileged Are You?*
- Screening for structural vulnerability
- Asking yourself, "What role might social, political, economic, or cultural factors play" in the health problems this patient is facing? (Metzl et al., 2018, p. 193)

Antecedents of Patient-Centered Care

Antecedents	Examples
1. Organizational Values, Policies, and Procedures	1.1 Health care provider skills and training (e.g., case management, motivational interviewing)<br>1.2 Creating environments that are safe, stable, and social<br>1.3 Deploying interprofessional care teams<br>1.4 Simplifying the logistics and continuity of access to providers<br>1.5 System rooted in harm reduction and the social determinants of health<br>1.6 Comprehensive assessment and screening procedures
2. Clinical Approaches that Strengthen Therapeutic Alliance	2.1 Open communication and active listening<br>2.2 Investing time to build trust<br>2.3 Affirming the client's ability to succeed in their goals<br>2.4 Adopting an individualized approach<br>2.5 Collaborating with clients<br>2.6 Taking a holistic view
3. Clinical Approaches that Support Shared Decision-Making	3.1 Sharing information in a manner appropriate for the client<br>3.2 Empowering clients as experts in treatment need and building capacity for self-responsibility<br>3.3 Establishing respectful relationships with clients<br>3.4 Being flexible in approaches offered
4. Clinical Approaches that Support Individualized Care	4.1 Encouraging clients' input and preferences<br>4.2 Establishing caring relationships with clients<br>4.3 Offering a flexible continuum of care

(Marchand, et al., 2019)

Structural Vulnerability Assessment Tool
(Bourgois et al., 2017)

Domain	Screening Questions
Financial security	Do you have enough money to live comfortably – pay rent, get food, pay utilities, telephone?
Residence	Do you have a safe, stable place to sleep and store your possessions?
Risk environments	Do the places where you spend your time each day feel safe and healthy?
Food access	Do you have adequate nutrition and access to healthy food?
Social network	Do you have friends, family, or other people who help you when you need it?
Legal status	Do you have any legal problems?
Education	Can you read?
Discrimination	Have you experienced discrimination?
Ask yourself (silently): May some service providers (including me) find it difficult to work with this patient?	

Integrated Approach to Primary Care: Eliminating Stigma in the Syndemic

- All patients
  - *Perform screening (OUD, HIV, HCV) according to evidence-based guidelines*
  - *Assess structural vulnerability*
- New opioid prescriptions – CDC Guidelines for Management of Chronic Pain
  - *Consider medication-alternatives to pain management*
  - *Prescribe naloxone along with opioid medications*
  - *Assess barriers patients face to safe use, storage, and disposal of prescription medications*
- Patients with opioid use disorder
  - *Obtain a DATA 2000 waiver to provide MAT*
  - *Perform regular assessments (HIV, HCV, structural barriers)*

Questions?

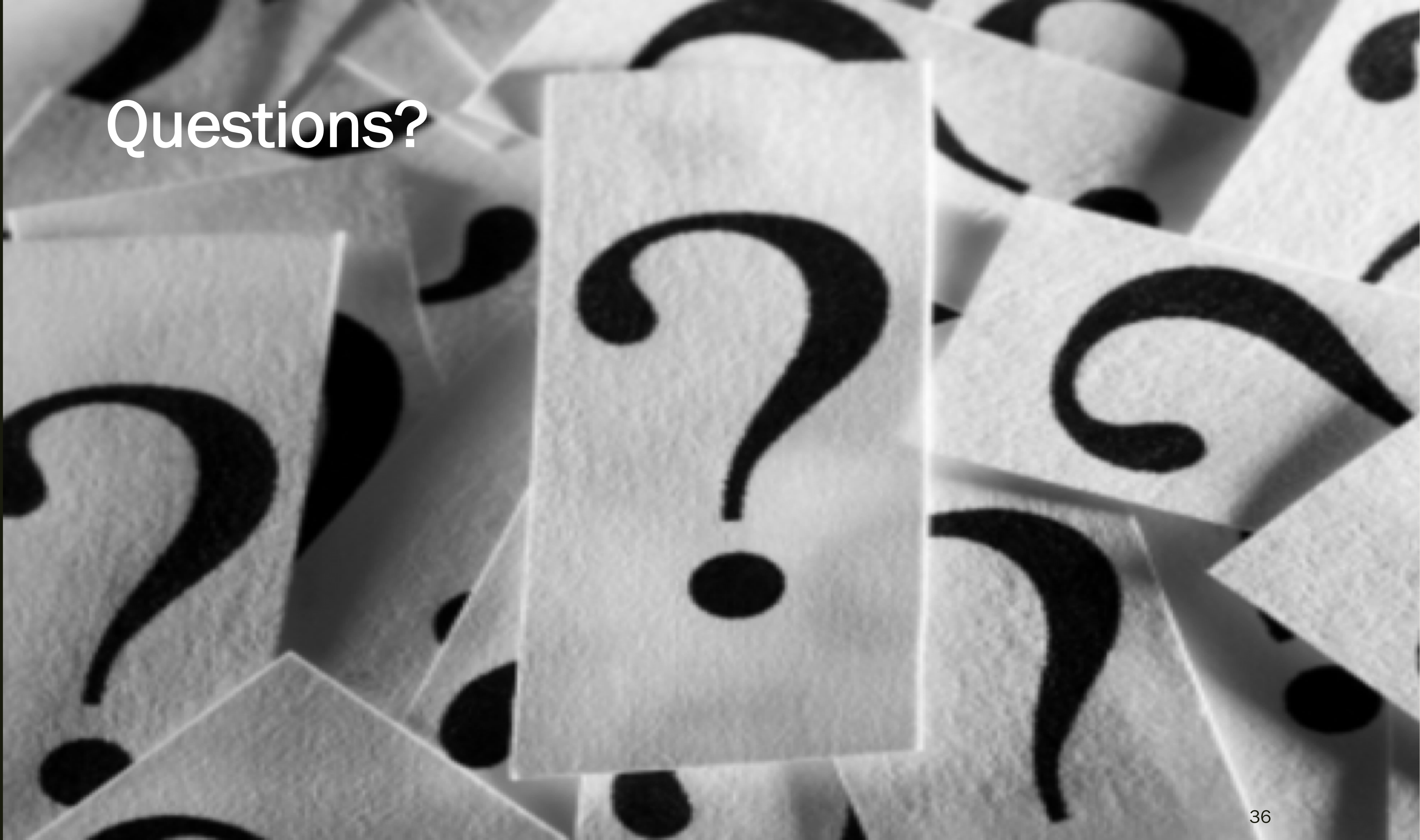

References

- Bourgois, P., Holms, S. M., Sue, K., & Quesada, J. (2017). Structural vulnerability: Operationalizing the concept to address health disparities in clinical care. *Academic Medicine, 92*(3), 299-307. doi: 10.1097/ACM.0000000000001294
- CDC/NCHS, National Vital Statistics System, Mortality. CDC WONDER, Atlanta, GA: US Department of Health and Human Services, CDC; 2018. https://wonder.cdc.gov.
- Grappone, G. (2017). Overcoming stigma. NAMI Advocate. https://www.namikenosha.org/uploads/4/9/0/6/49066629/overcoming_stigma.pdf.
- Link, B. & Phelan, J. (2001). Conceptualizing stigma. *Annual Review of Sociology, 27*, 363-385.
- Hansen, H. & Metzel, J. M. (2019). *Structural competency in mental health and medicine: A case-based approach to treating the social determinants of health.* Springer.
- Marchand K, Beaumont S, Westfall J, et al. Conceptualizing patient—entered care for substance use disorder treatment: findings from a systematic scoping review. Subst Abuse Treat Prev Policy. 2019;14(1):37 doi: 10.1186/s13011-019-0227-0
- Mathers, B., Degenhardt, L., Phillips, B., Wiessing, P. B., Hickman, M., Strathdee, S. A, …, 2007 Reference Group to the UN on HIV and Injecting Drug Use. (2008). Global epidemiology of injecting drug use and HIV among people who inject drugs: A systematic review. The Lancet, 372(9651), 1733-1745. doi: 10.1016/S0140-6736(08)61311-2
- Metzel, J. M. & Hansen, H. (2014). Structural competency: Theorizing a new medical engagement with stigma and inequality. *Social Science & Medicine, 103*, 126-133. Doi: 10.1016/j.socscimed.2013.06.032
- Metzl, J. M., Petty, J., Olowojoba, O. V. (2018). Using a structural competency framework to teach structural racism in pre-health education. *Social Science & Medicine, 199*, 189-201.
- Meyerson, B., Barnes, P., Emetu, R., Bailey, M., Ohmit, A., & Gillespie, A. (2014). Institutional and structural barriers to HIV testing: Elements for a theoretical framework. *AIDS Patient Care & STDs, 28*(1), 22-27. Doi: 10.1089/apc.2013.0238
- Misir, P. (2015). Structuration theory: A conceptual framework for HIV/AIDS stigma. *Journal of the International Association of Physicians in AIDS Care, 14*(4), 328-334. Doi: 10.1177/2325957412463072
- Nelson, P. K., Mathers, B. M., Cowie, B., Hagan, H., Des Jarlais, Horyniak, D., & Degenhardt, L. (2011). Global epidemiology of hepatitis B and hepatitis C in people who inject drugs: Results of systematic reviews. *The Lancet, 378*(9791), 571-583. doi: 10.1016/S0140-6736(11)61097-0
- Nicholson, A. (2018). *Integrating responses at the intersection of opioid use disorder and infectious disease epidemics: Proceedings of a workshop*. Washington, DC: The National Academies Press.
- Perlman, D. C. & Jordan, A. E. (2018). The syndemic of opioid misuse, overdoes, HCV, and HIV: Structural-level causes and interventions. *Current HIV/AIDS Reports, 15*(2), 96-112. Doi: 10.1007/s11904-018-0390-3
- Peters, P. J., Pontones, P., Hoover, K. W., Patel, M. R., Galang, R. R., Shields, J., …, Indiana HIV Outbreak Investigation Team. (2016). HIV infection linked to injection use of oxymorphone in Indiana, 2014-2015. *New England Journal of Medicine, 375*(3), 229-239. doi: 10.1056/NEJMoa1515195
- Singer M. & Clair S. (2003). Syndemics and public health: reconceptualizing disease in bio-social context. *Medical Anthropology Quarterly, 17*(4), 423–41. doi: 10.1525/maq.2003.17.4.423
- Tervalon, M. & Murray-Garcia, J. (1998). Cultural humility versus cultural competence: A critical distinction in defining physician training outcomes in multicultural education. *Journal of Health Care for the Poor and Underserved, 9*(2), 117-125. doi: 10.1353/hpu.2010.0233
- Van Handel, M. M., Rose, C. E., Hallisey, E. J., Kolling, J. L., Zibbell, J. E., Lewis, B., …, Brooks, J. T. (2016). County-level vulnerability assessment for rapid dissemination of HIV or HC infections among persons who inject drugs, United States. *Journal of Acquired Immune Deficiency Syndrome, 73*(3), 323-331.
